# Green synthesis of capped gold nanoparticles and their effect on Gram-positive and Gram-negative bacteria

**DOI:** 10.4155/fsoa-2017-0062

**Published:** 2017-09-05

**Authors:** Yashvant Rao, Gajendra K Inwati, Man Singh

**Affiliations:** 1 Centre for Nanosciences, Central University of Gujarat, Gandhinagar, Gujarat, India; 2 School of Chemical Sciences, Central University of Gujarat, Gandhinagar, Gujarat, India

**Keywords:** AFM, antibacterial, FTIR, MIC, nanomedicines, natural synthesis, TEM

## Abstract

**Aim:**

We report synthesis of capped gold nanoparticles (C-AuNPs) of ≈20–30 nm by reducing HAuCl_4_ with flower and leaf extracts of *Ocimum tenuiflorum*, leaves of *Azadirachta indica* and *Mentha spicata* and peel of *Citrus sinensis* plants.

**Methods:**

Atomic force microscopy (AFM) and transmission electron microscopy (TEM) determined their size, shape and topographical structures. The C-AuNPs with UV-Vis spectrophotometer produced a maximum absorption within 530–535 nm wavelengths. Their Fourier transform IR stretching frequencies, from 450 to 4000 cm^-1^, have inferred HAuCl_4_ reduction to Au.

**Results:**

The 512 and 600 μgml^-1^ C-AuNP MICs were expressed on antimicrobial strains *Staphylococcus aureus, Pseudomonas aeruginosa* and *Klebsiella pneumoniae*, respectively.

**Conclusion:**

The chosen plant extracts have reduced the Au^3+^ to Au^0^ with simultaneous *in situ* capping with bacteria inhibiting activities. Green routes for C-AuNP synthesis could be an asset for several other biomedical and bioengineering applications.

## Lay abstract

*Staphylococcus aureus, Pseudomonas aeruginosa* and *Klebsiella pneumoniae* are pathogenic bacteria that can be very dangerous for humans. Their growth is inhibited by gold nanoparticles (AuNPs) capped with chemical agents from the flowers and leaves of *Ocimum tenuiflorum*, leaves of *Azadirachta indica* and *Mentha spicata* and peel of *Citrus sinensis*. Capped AuNPs expressed higher antibacterial activities as compared with Ag, Cu and Se metallic nanoparticles obtained by chemical reductions contrary to green routes. Capped AuNPs synthesized through green routes could also be an asset for several other biomedical applications.

Scientific interest into use of natural resources to synthesize metallic nanoparticles (NPs) has been rapidly growing, especially concerning Cu, Ag and Au NPs as biocompatible nanomaterials. Metallic NPs expressing antimicrobial activities are being widely applied in several fields of nanoscience and nanotechnology. The use of these nanomaterials could supersede the activities of routinely used medicines [1–3], along with new industrially useful nanoformulations. Capped gold nanoparticles (C-AuNPs) are extensively used as an efficiently refined catalyst for medical and gene therapies, and for diagnostic, biomedical and biological purposes [4–6]. In these applications, C-AuNPs of ≤50 nm size have been preferred for effective uses. Despite wider applications, the synthetic methods for C-AuNPs are resource consuming with a multistep processes and there is a need to develop a new synthetic route with natural resources to achieve their desired sizes. Therefore, it was our aim to prepare C-AuNPs of ≈20–30 nm stable for longer durations.

The main advantage of C-AuNP synthesis by plant extracts (PE) is very low toxicity as compared with those obtained via routine chemical reduction. For such considerations, several synthesis techniques have been applied to generate C-AuNPs of various geometrical dimensions as well as to functionalize their surfaces to further widen their functionality and applications [7–10]. Contrary to this, for controlling the size and shape of C-AuNPs, various reducing agents, stabilizers and solvents have been applied for the C-AuNPs preparation [11,12]. Reportedly, several researchers have worked toward C-AuNP synthesis by using PEs but could not synthesize the C-AuNPs of ≈20–30 nm and ≈100% purity with longer stability [12]. Hence, it is always advisable to increase the database of reducing natural [13] resources for synthesis and size control of C-AuNPs with efficient reducing resources, preferably PEs. Our one-step approach for achieving ≈100% pure gold nanoparticles (AuNPs) of ≈20–30 nm aimed to provide an easier method.

PEs have several reducing chemical structures (Supplementary Figure 1) along with capping agents.

C-AuNPs exhibit strong absorption spectra in the visible range, which is due to the coherent oscillations of free electrons on the surface of C-AuNPs, which can be applied to surface plasmon resonance (SPR). Currently, SPR spectra of C-AuNPs have immense applications and our process could be of interest [14,15] for such surface sciences. The C-AuNPs are in close contact with chemical agents that contains the enzyme antioxidants [13] and medicinal components [16].

Synthetic and surface modification methodologies play a critical role in developing the physicochemical, electrical and optical properties to enhance C-AuNP efficiency. Metals such as copper have been used to kill microbes, but C-AuNPs have been only slightly explored for antibacterial studies [17,18]. C-AuNPs have been largely used as a catalyst in the last few decades [19,20]. They have also been used to kill pathogenic bacteria in treatments of arthritis [21,22]. Medical applications can also include a use of sulfur–gold compounds to control anti-inflammatory [23] diseases. C-AuNPs inhibit proliferation of T cells by modifying permeability of the mitochondrial membrane [24,25], along with effective killing of bacteria. The specific mechanism of C-AuNPs, due to their hydrophobicity owing to use of PE, attacks cell walls of Gram-positive and Gram-negative bacteria. Reportedly, the C-AuNPs of ≈20–30 nm size limit the enzymatic activity of liposome in macrophages [26] due to cellular organelles.

Use of C-AuNPs in a reasonable amount do not negatively affect human cells [27–29]. Thus, we have explored the Au-supported antibacterial activity for Gram-positive and Gram-negative bacteria (Table 1). *Staphylococcus aureus* is a Gram-positive bacteria, found in the nose, respiratory system and on the skin. *Pseudomonas aeruginosa* is Gram-negative and causes disease in plants, animals and human, due to its bactericidal toxic activities. C-AuNPs have destroyed pathogenicity of these bacteria. Thereby we have studied the antibacterial effects of C-AuNPs with *S. aureus, P. aeruginosa* and *Klebsiella pneumoniae*. We have further extended our studies to the pathogenic bacterial growth to analyze and determine the MIC to express the unique potential of C-AuNPs for bioactive genesis. The characterization of C-AuNPs was made with transmission electron microscopy (TEM), atomic force microscopy (AFM), dynamic light scattering (DLS), UV-Vis and Fourier transform IR (FTIR) with definite cohesive as well as kinetic forces. These studies furnish adequate information about C-AuNPs for interaction and killing mechanisms of Gram-positive and Gram-negative bacteria along with their green synthesis.

**Table 1.** **Plant parts used for capped gold nanoparticle synthesis and pathogenic bacteria with their origin.**

**Sr. No.**	**Plants**	**Plant parts for C-AuNP synthesis**
1.	*Ocimum tenuiflorum*	Flowers and leaves

2.	*Azadirachta indica*	Flower

3.	*Mentha spicata*	Leaves

4.	*Citrus sinensis*	Peel

^C-AuNP: Capped gold nanoparticle.^

## Experimental procedure

### Collection & processing of material for C-AuNP synthesis

PEs were prepared from dried plant parts collected locally. The chemical components present in flower and leaves of Basil, flowers of Neem, leaves of Mentha and peel of orange fruit extracts have acted as the reducing agent for HAuCl_4_ to Au^0^. The HAuCl_4_, 99.5%, (Sigma-Aldrich, Bangalore, India), and other chemicals of analytical grade were used without further purification. The 1.0 mM HAuCl_4_ aqueous solution was prepared using Millipore water of 5 μcm^-1^. The bacteria *S. aureus* (NCIM 2079), *P. aeruginosa* (NCIM 2036) and *K. pneumoniae* (NCIM 2719) were collected from National Collection of Industrial Microorganisms (NCIM), National Chemical Laboratory, Pune, India. The media used for bacteria *S. aureus, P. aeruginosa* and *K. pneumoniae* have the nutrient broth from Hi-media, which contains NaCl 5.0%, Beef extracts 10.0% and Peptone 10.0% (pH 7.2) composition (μgml^-1^). In this method, AuNPs were synthesized by a single-step process at room temperature (RT). For this purpose, biological resources (hazardless) were used for AuNPs synthesis. Their size of ≈20–30 nm was confirmed by TEM and AFM. The stability and particle distribution were optimized by UV-Vis and DLS.

## Methods

**Figure 1.**
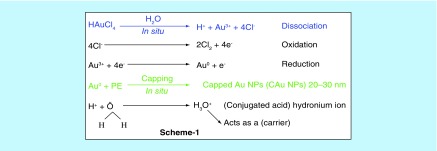
**Chemical reaction mechanism.**

**Figure 2.**
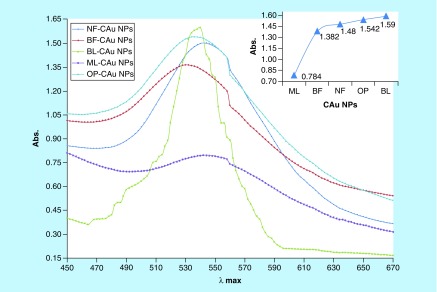
**UV-Vis spectroscopy depicts maximum absorbance within 530–535 nm.**

Approximately 100 g each of flowers, leaves and peel were separately washed 3–4 times with tap water followed with Millipore water for preparing PEs. The plant parts were separately dried at RT for 24–48 h in the dark followed by dicing in a blade blender until a homogeneous powder was obtained. No metallic part was reduced from the blender which was checked with the wet chemical analysis. The 0.50 g powder of each plant part was dissolved in 50.00 ml Millipore water weight/volume separately. Each mixture was centrifuged thrice at 7500 r.p.m. for 5, 10 and 15 min. For each time slot, the supernatants were collected and filtered twice through Whatmann filter paper (G4) at normal temperature and pressure. The filtrate was kept in neat and clean sample vials, which were rinsed twice with absolute alcohol. The filtrates in vials were kept at -18°C until a use for C-AuNP synthesis. These extracts had various reducing and stabilizing chemical compounds, which have reduced the Au^3+^ to C-AuNPs (Figure 1). Gold ion (Au^+3^) solution was prepared with 1.0 mM HAuCl_4_ solution in Millipore water by mixing 10 ml (1.0 mM HAuCl_4_) with 90 ml (volume/volume) (1:9) of PEs, respectively. The resultant solution was magnetically stirred at 350 r.p.m. for 15 min to homogenize the shape and size of C-AuNPs. The original color of PEs changed to ruby red, which has been an initial indication for C-AuNPs formation. It was further verified by UV-Vis absorption peak at 535 nm (Figure 2).

Reportedly, the PEs contain several functional chemical groups like acid (Supplementary Figure 1), which assisted the reduction reaction of Au^3+^ to Au^0^ in H_2_O (*in situ*) is expressed in Figure 1. Since the Au^0^ has a natural tendency to coagulate, thus the chemical agents released out of PEs had capped the Au^0^ *in situ* and stabilized the size of AuNPs ≈20–30 nm. The PEs have valuable chemical agents with several functional groups, which substantially cap the AuNPs along with inducing additional activities in C-AuNPs (Supplementary Figure 1). This is an *in situ* capping which is explained in the chemical reactions in Figure 1. H_3_O^+^ has a kinetically active and electron deficient site, and disperses the chemical species of PE for chemical capping. Contrary to the H^+^, which is also furnished by HAuCl_4_ on dissociation, the chemical compounds present in Basil Extracts (BE) had also furnished the H^+^. Thus, due to a common ion effect, the release of 4e^-^ from 4Cl^-^ on its oxidation to 2Cl_2_ could be influenced. These 4e^-^, in turn, had reduced the Au^3+^ to C-AuNPs in two steps (Figure 1). After a reaction of Au^+3^, the 1e^-^ is still left unused. However, the same mechanism could have been catalyzed by hydronium ion (H_3_O^+^) as it could act as an e^-^ carrier in a process of Au^3+^ reduction. The electrons released from oxidation step could be delayed but a release of H^+^ from the BE may be spontaneous as the H_2_O quickly receives H^+^ on forming the H_3_O^+^ (Figure 1). Thus, an excess of H^+^ on getting released by the BE could facilitate the HAuCl_4_ reduction. Along with the above mechanism, there is one more possibility that the one e^-^ could be utilized by the H^+^, which is released on the HAuCl_4_ dissociation step. The already available H^+^ through the H_3_O^+^ could combine with the Cl^-^ on disruption of the functional group in the PE and get engaged in forming the Au^3+^ to Au^0^ on 2H^+^ + 2e^-^ → H_2_. The reaction mechanism for releasing the H^+^ from the BE is supported by H_2_O, which acts as a conjugate base, an acid pair on modifying the condition for the common ion effect. So, the HAuCl_4_ dissociation is catalyzed into its components. However, an excess of Cl^-^ could also be involved in reduction reaction as no other source of the H^+^ is available with orange peel extract. Hence, the H^+^, which is released from the HAuCl_4_, seems responsible for Au^3+^ to C-AuNPs. Therefore, considering the above-mentioned overall redox mechanism, it is rare that the plant sources efficiently substitute the redox mechanism to achieve the desired state of the metal. In our studies, the reduction oxidation is remarkably achieved through the chemical agents via the chosen precursor facilitated by the plant resources of electron release and consumption. The conceptual illustrations are depicted in Figure 1. The various PE agents are capped with AuNPs with stronger Van der Waals forces and the unoccupied forces of capped agents interact with *S. aureus, P. aeruginosa* and *K. pneumoniae* bacteria. This mechanism of C-AuNPs either immobilizes or disrupts the base pair of DNA through interaction mechanism. The C-AuNPs act as a core for holding the antibacterial chemical agents of PE with selective binding activities. For an antibacterial study, a stock solution (1024 μgml^-1^) of C-AuNPs was prepared in Millipore water for each PE. The dilutions were prepared with microbiological media, which were used for bacterial serum NCIM 2079, NCIM 2036 and NCIM 2719 with NaCl, Beef extract and Peptone (pH 7.2) [30], respectively. The 64–1024 μgml^-1^ C-AuNPs were added to a culture medium for antibacterial study with pathogenic bacteria. The bacterial serum and C-AuNPs with cultures at 37°C kept in an incubator for overnight.

## Results & discussion

### Visual observation

The visual observation for C-AuNP synthesis was obtained on getting a shining orange and ruby red color of colloidal solutions, which indicate C-AuNP formation. The advantages for a use of PEs include decreasing the size of C-AuNPs along with rapidly capping with the chemical agents of PEs. On increasing a rate of reduction reaction [31], the rapid reaction provided a uniform growth condition, leading to homogenization and capping the C-AuNPs into smaller sizes, determined with adequate devices.

### UV-Vis analysis

The characterization with UV-Vis has identified the C-AuNPs. Figure 2 shows the UV-Vis spectra for C-AuNPs, which were developed in the aqueous PE, out of 1.0 mM of HAuCl_4_. From 200 to 800 nm (?) spectral range, the absorption peaks are seen but the C-AuNPs samples obtained with different PEs have shown a distinct and maximum absorbance at  approximately 535 nm [32,33]. This peak has indicated the C-AuNPs formation and a broad peak at 535 nm [32,33] had revealed a dispersion of C-AuNPs in medium. The stability of freshly prepared C-AuNPs was determined by comparing their UV-Vis spectra against the stored C-AuNPs for more than 6 months at RT. Stability of C-AuNPs for a longer time infers not only the C-AuNPs homogenous dispersion but also their capping with the moderate Van der Waals forces. The Au surface area interacts with chemical compounds of PEs through the residual forces to prevent agglomeration. Some of the PE components have adequately capped the AuNPs whose stability for longer time with higher thermodynamic and kinetic stability is seen. This is another advantage of C-AuNPs with PEs, where a sharper λmax is seen as compared with C-AuNPs and indicates an adequate capping. The capping has depicted an effective distribution and most optically active nanomaterials. The variations in λmax values and intensities for C-AuNPs with basil flower (BF), basil leaves (BL), neem flower (NF), mentha leaves (ML) and orange peel (OP)-C-AuNPs prove that C-AuNPs had different chemical compounds with different responses. For example, a broader λmax had found a mild response, with the C-AuNPs which shows the h*ν* interaction with C-AuNPs, where it could not have detained the larger UV light. Since no blue or red shifts are seen in Figure 2, this inferred a similar order and mechanism of PE. The UV response provides a database to choose the PEs for developing the UV sensitive C-AuNPs or UV least sensitive C-AuNPs.

### FT–IR analysis

**Figure 3.**
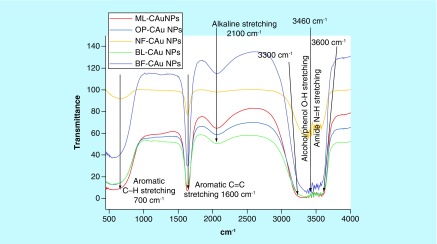
**Stretching frequency at 700 cm^-1^ shows aromatic C–H bending, 1600 cm^-1^ aromatic C=C bending, 2100 cm^-1^ alkyl C=C stretching, 3300–3460 cm^-1^ alcohol/phenol O–H and 3600 cm^-1^ amide N-H.**

The PEs have certain functional moieties that probably with п conjugation could cause a sharper UV response. The potential functional groups responsible for C-AuNPs stabilization were analyzed with FTIR (Figure 3). The C-AuNPs synthesized with PEs could contain functional groups, which might have played an important role in C-AuNPs stabilization during a synthetic process. The FTIR spectra (Figure 3) have shown a presence of five major transmission stretching frequency peaks from 3700–3500, 3460–3300, 2100, 1700–1500 and 860–680 cm^-1^. The first two stretching peaks are attributed to amide N-H stretching vibration from 3300 to 3460 cm^-1^. The second belongs to the alcohol/phenol [34] O–H stretching vibration at 3600 cm^-1^. The other three absorption peaks represent an availability of alkynyl C≡C stretching at 2100 cm^-1^, aromatic C=C bending at 1600 cm^-1^ and aromatic C–H bending at 700 cm^-1^ vibrations, C=C stretching vibration of alkyl and C=C, C–H aromatic bending vibration, respectively. These functional groups have played an important role in C-AuNP synthesis. Of course, amide N-H stretching, alcohol/phenol O–H bond stretching, alkyl C=C stretching, aromatic C=C bending and aromatic C–H bending are important for their shape, size, morphology and surface properties with functional groups. Figure 3 depicts the source of listed functional groups. It was supposed to separate out each functional component from each PE, but the purpose of our study was to synthesize the C-AuNPs of ≈20–30 nm size and to check their thermodynamic stability.

### DLS analysis

**Figure 4.**
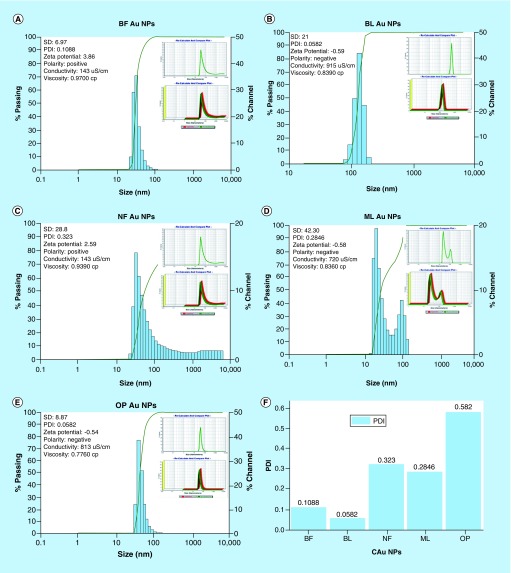
**DLS micrograph of C-Au NPs. DLS images of C-AuNPs from PE (A) and (B) C-AuNPs with extracts BF and BL (C) NF C-AuNPs (D) ML C-AuNPs (E) OP C-AuNPs fruit respectively and (F) depicts PDI values of C-AuNPs.** BF: Basil flower; BL: Basil leaf; C-AUNP: Capped gold nanoparticle; DLS: Dynamic light scattering; ML: Mentha leaves; NF: Neem leaves; NP: Nanoparticle; OP: Orange peel; PE: Plant extract.

FTIR spectra (Figure 3) have inferred a presence of functional groups that might have helped the C-AuNP distribution in the chosen medium. DLS was determined thrice and calculated an average size, and polydispersity index (PDI) values of AuNPs of ≈20–25 nm capped with BF, BL, NF, ML and OP are 0.1088, ≈30 nm 0.0582, ≈25–30 nm 0.323, ≈25–30 nm 0.2846 and 20–30 nm 0.0582, respectively (Figure 4A–E). These PDI values indicate that the capping energy remains different for each PE. Thus, ultimately the capping with different PEs causes the different impacts on antibacterial activities of C-AuNPs. Consequently, the stability of C-AuNPs stored for >6 months was confirmed by ζ-potential, PDI and size.

### TEM analysis

**Figure 5.**
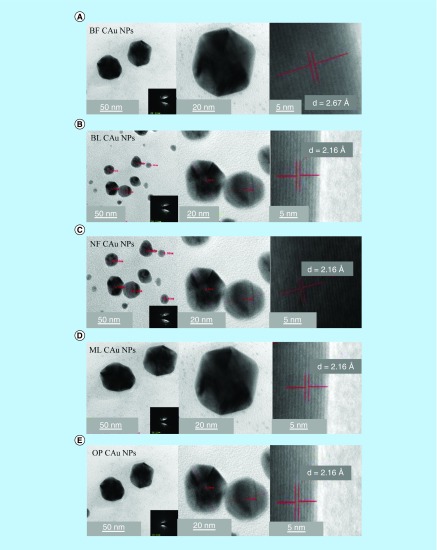
**TEM image of C-AuNPs.** **(A)** and **(B)** C-AuNPs with extracts of flower and leaves of BF and BL **(C)** NF C-AuNPs **(D)** ML C-AuNPs **(E)** OP C-AuNPs fruit, respectively. Every graph has four images with different pixels and taken from 50, 20 and 5 nm with SAED pattern. C-AuNPs were analyzed with TEM and found <20 nm of size with 0.267 and 0.216 nm d spacing. BF: Basil flowers; BL: Basil leaves; C-AuNP: Capped gold nanoparticle; ML: Mentha leaves; NF: Neem leaves; NP: Nanoparticle; OP: Orange peel; SAED: Selected area electron diffraction; TEM: Transmission electron microscopy.

TEM micrographs for C-AuNPs have depicted a spherical shape of ≤20 nm in diameter (Figure 5A). The C-AuNPs were analyzed at 300 kV as an accelerating voltage that indicated a magnification of 75 kx. The C-AuNPs capped with BF, BL, NF, ML and OP were analyzed with a 50–5.0 nm scan area 20.94, 18.54 nm; 20.94, 18.58 nm; 15.80, 20 nm; 24.94, 21.56 nm; and 21.66, 26.26 nm of diameter in size of spherical morphology on a crystalline scale, respectively. The interspacing distances (d) are 2.16 and 2.67 Å, which infer a difference in capping [35].

**Figure 6.**
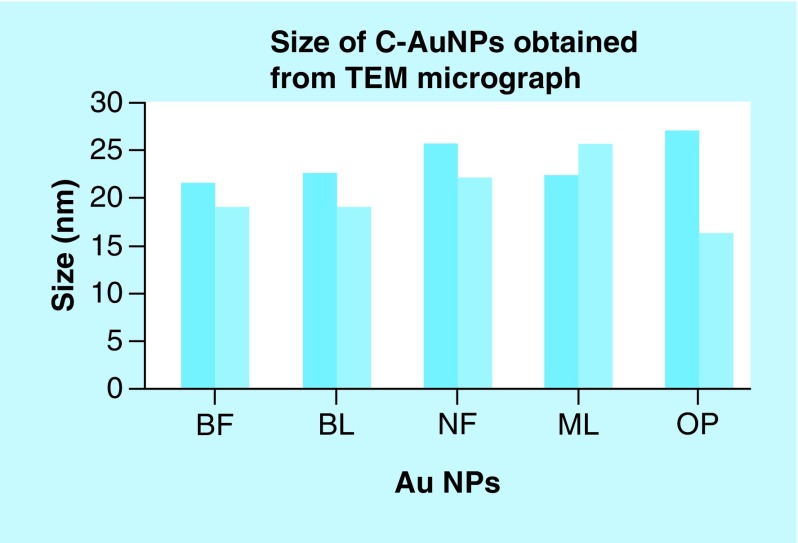
**Nanoparticle size analyzed by TEM.** NP: Nanoparticle; TEM: Transmission electron microscopy.

The level of C-AuNPs with PEs is higher (Figure 6) as compared with the media prepared with deionized water (DI) water (Figure 5A–E). This might have been expected since the media contains the nutrients as well as free ions where they could be exchanged with the PEs on C-AuNPs surfaces causing aggregation. Such an exchange process unlikely could occur when the media molecules have high molecular weight, used for surface modification [36]. The single crystalline nature of biogenic spherical C-AuNPs is reflected in the hexagonal nature of selected area electron diffraction spots (Figure 5A–E) and corresponding entire spherical area shown in Figure 5 at 20 nm scale. The diffraction spots could be indexed on the basis of the fcc structure of C-AuNPs and are assigned to [111, 36], Bragg reflections with a lattice spacing of 2.16, and 2.67 Å. Spherical NPs are highly [111] oriented with the top surface normal to electron beam [5,35]. The spherical C-AuNPs could be seen as the shape is visible. Topographic size and height analysis of each is shown in Figure 6.

### AFM analysis

**Figure 7.**
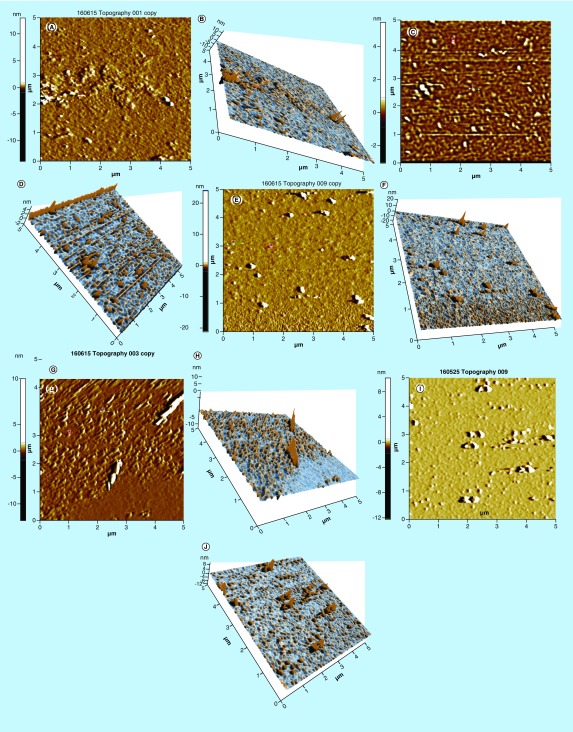
**AFM topographical image.** AFM micrograph of C-AuNPs. **(A–J)** depicting shape, size, height, roughness and 3D topographical image due to natural properties of PEs. **(A & B)** and **(C & D)** graphical representation of morphology, size and 3D topographical image of C-AuNPs from extracts of flower and leaves of *Ocimum tenuiflorum*; **(E & F)** flower extract of *Azadirachta indica*; **(G & H)** leaves extract of *Mentha spicata*; and **(I & J)** peel extract of *Citrus sinensis* fruit, respectively. Every graph shows different size and morphology with a histogram of C-AuNPs. C-AuNP: Capped gold nanoparticle; PE: Plant extract.

For determining topographical morphology, size and surface properties of C-AuNPs with AFM, an aqueous C-AuNPs solution was spreaded on cleaned mica sheets of 20 × 20 mm^2^ size, which was glued to metal pads for stabilizing C-AuNPs solution. The colloidal solution was deposited on mica sheet for 5 min at RT followed by three- to four-times washing with the dissolved liquid. The X, Y and Z in high range and the Z scanner range is 9.9 Å. Silicon cantilevers (Nchr) with 30 nm thick aluminum reflex coating was used. According to the producer's data sheet, the cantilever spring constant was of the 1.5–15 nm range with resonance frequency of 150 ± 75 kHz, the tip radius was <10 nm. The analysis of C-AuNPs has a size as well as 3D topography shown in Figure 7A–E.

The use of incoherent light experiments, performed in a vicinity of SPR frequency, measured a phase relaxation time and nonlinear susceptibility of C-AuNPs of 20–30 nm. The 3D topography morphology represents an enhanced color of maximum height of BF-C-AuNPs, which showed a particle of size of 20.24 nm and roughness of two selected particles of 1.91 and 1.95 Å. The BL-C-AuNPs showed a size of 24.0 nm and the 2.05 and 2.38 Å roughness of selected particle. Similarly, the NF-C-AuNPs showed of size 27.0 nm and roughness of 3.86 and 3.71 Å. The ML-C-AuNPs showed a size of 25.0 nm and roughness of 3.09 and 2.45 Å. The OP-C-AuNPs showed a size 30.0 nm, and the roughness of particles were 2.83 and 3.22 Å, as shown in the topographical image [37,38]. AFM micrograph of C-AuNPs is shown in Figure 7, which is repeatedly seen for a large fraction of nanotriangles (Figure 2). A topographic size and height analysis along with the triangle indicated by a line in along the image is shown in the bottom part of Figure 7A–J. The particle is of ≈20–30 nm size and is quite smooth (very rare roughness) over the surface. The electron diffraction analysis of C-AuNPs (Figure 5) has indicated that the C-AuNPs are of [111] orientation. Figure 5 shows the TEM image of vertex of spherical form. The lattice spacing for the lattice planes is 2.16 Å and is parallel to hexagonal edges of C-AuNPs.

## Antibacterial study of AuNPs capped with BF, BL, NF, ML & OP extracts

### MIC analysis

MIC is the lowest drug concentration that inhibits a visible growth of an organism on an overnight incubation. This period could be extended for organisms, such as anaerobes, and for prolonged incubation for growth with BF, BL, NF, ML and OP-C-AuNPs. The ‘Au standard’ for determining susceptibility of organisms to antimicrobial is used. These activities assess a performance of other methods for the purpose of susceptibility testing. The MIC depicts an unusual resistance, to give a definitive result of testing methods. The agar dilution method as an amended version of the procedure has been described in the BSAC Guide to Sensitivity Testing. The test can be adapted for determining a minimum bactericidal concentration [39,36,37] for an antibacterial activity of an organism by substituting composition and then subculturing to drug-free media truncated for a use as breakpoint method. Bactericide properties of C-AuNPs find the surviving cells, which formed the colonies after 120 min in culture media. After 2 h, the experiment was initiated, where the highest bacterial growth is noted. In this experiment the growth was inhibited ≥512 μgml^-1^. The C-AuNPs inhibited a growth of *S. aureus, P. aeruginosa* and *K. pneumoniae*, thus the AuNPs acted as a better bactericide than other nanomaterials, which are in use to-date. Therefore, our C-AuNPs could become the new standard nanomaterials for this purpose. Their better dispersion and roughness have enhanced their bactericide activities. C-AuNP bactericidal activities were compared with mordenite clinoptilolite and found to be more efficient at killing *S. aureus, P. aeruginosa* and *K. pneumoniae* at ≥512 μgml^-1^. In the context of interfacial chemical activities, the C-AuNPs, with an adequate element of hydrophobicity, generate an affinity toward Gram-positive and Gram-negative bacteria as their cell wall is made up of peptidoglycan with a suitable hydrophobicity in a structural orientation. Thus, the capping materials that were sourced from the PEs have been effective at helping to develop interaction with the cell wall materials of both the bacterial types. Thus, the structurally active chemical process initiates a Gibbs energy change to occur. Such energy changes with Le Chatelier concept lead to approach or allow the C-AuNPs to enter the cell and intercalate the bacterial DNA.  Structurally active chemical activities that have been operational because of a constitutional make up of PEs with AuNPs *vis-à-vis* Gram-positive and Gram-negative bacteria could become part of a new database. Such a database could be extended to medical treatment as well as to nanomedicines for industrial use, Swiss cheese production and so on. Thus, the interfacing and chemical coordinates of C-AuNPs have authentically inhibited the growth of Gram-positive and Gram-negative bacteria at 512–600 μgml^-1^, which were able to kill the selected pathogenic bacteria.

For antimicrobial activities, several approaches, such as disc or well diffusion, are in use where the positive control is an essential condition that is not needed for MIC determination. Contrary to the above, our purpose was to ensure expression of antibacterial activities of C-AuNP, so we conducted the experiments for a coagulation of bacterial cells that were dispersed in culture media. Such a mechanism acted as an indicator for cell death. Therefore, the doses of C-AuNPs to coagulate the bacterial cells from dispersion were considered to determine the MIC. We repeated the coagulation experiments with specified dose of C-AuNPs at several intervals, which produced the similar coagulation results that authentically determined the antibacterial activities.

**Figure 8.**
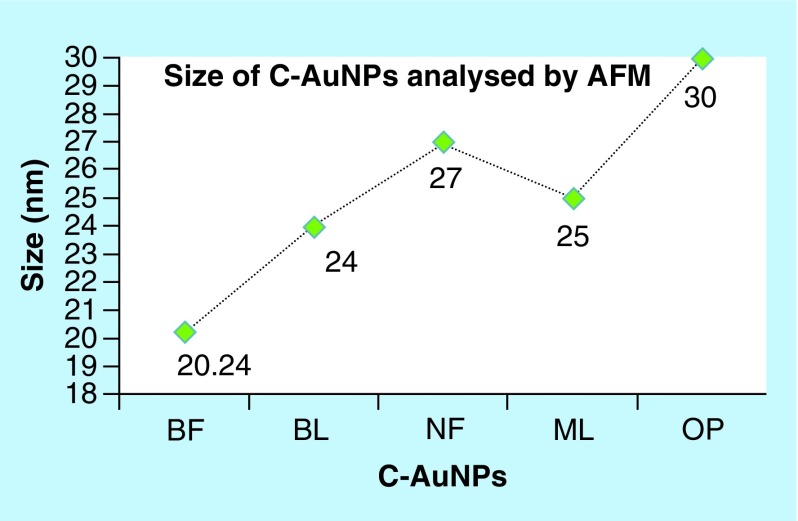
**Size of gold nanoparticles capped with BF, BL, NF, ML and OP analyzed by AFM.** This figure depicts an effective C-AuNPs distribution with almost nil coagulation. AFM: Atomic force microscopy; AuNP: Gold nanoparticle; BF: Basil flowers; BL: Basil leaves; ML: Mentha leaves; NF: Neem flowers; OP: Orange peel.

**Figure 9.**
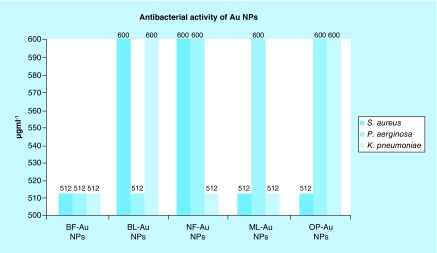
**Antibacterial activities of MIC values of gold nanoparticles capped with BF, BL, NF, ML and OP for Gram-positive and Gram-negative bacteria inhibit the *Staphylococcus aureus, Pseudomonas aeruginosa* and *Klebsiella pneumoniae*.** BF: Basil flowers; BL: Basil leaves; ML: Mentha leaves; NF: Neem leaves; NP: Nanoparticle OP: Orange peel.

The 512 and 600 μgml^-1^ C-AuNPs killed the pathogenic bacteria. The C-AuNPs as an efficient bactericide destroyed the bacteria due to their surface properties. The C-AuNPs killed pathogenic bacteria due to the cell wall interaction of C-AuNPs and by developing their interaction with bacterial toxin. Such interacting abilities of C-AuNPs with certain electrostatic force of attraction have enabled DLS analysis. The DLS analysis infers a functional interface between the C-AuNPs and the cell wall of bacteria where the C-AuNPs produced the reactive oxygen species, mainly ^.^OH and ^.^O_2_, which could destroy the bacterial cell wall. The mode of interactions of C-AuNPs on both Gram-positive and Gram-negative bacterial pathogens was studied at the MIC level. Therefore, the bacterial cultures were exposed to 1 ml of 512 and 600 μgml^-1^ C-AuNPs for 8h and stained with negative staining. Bacterial cultures treated with C-AuNPs have increased conductivity [40]. This could be well attributed to dissolution of cellular contents in culture broth, by a disruption of cell membrane structures with a loss of membrane permeability or an inability to sustain with the ATP production, necessary for maintaining the membrane dynamics. Also, a presence of phytochemicals [34] in PEs was believed to be responsible for AuNP formation [41]. Several doses of C-AuNPs have been applied for their antibacterial activities on Gram-positive and Gram-negative bacteria to determine the successful chemical initiation between the hydrophobicity of C-AuNPs and the peptidoglycan of bacterial cell wall. Therefore, out of 64, 128, 256, 512, 600, 700, 800, 900 and 1024 μgml^-1^ doses used for optimum inhibition of bacterial growth (whose details are given in Supplementary Tables 3, 4 & 5), the 512 and 600 μgml^-1^ have expressed maximum inhibition of bacterial growth. Therefore, these two concentrations are noted as the MIC. Thereby, the capping constituents outsourced from PEs for involving 5d^10^4f^14^6s^1^ electronic configuration of AuNPs on establishing a stronger binding due to their larger functional surface area noted as C-AuNPs. This could have definite chemical activities via stoichiometric ratio for the bacterial peptidoglycan extended to their DNA. Such intrinsic abilities of C-AuNPs and bacterial materials cause a maximum bacterial killing from 512 to 600 μgml^-1^ out of 64 to 1024 μgml^-1^. The activities versus concentration data are given in Supplementary Tables 3, 4 & 5. The C-AuNPs cause an activation of the protein surface of *S. aureus, P. aeruginosa* and *K. pneumoniae* bacterial cell by interaction through an antibacterial functional group, which decreases permeability of the cell membrane. The MIC values vary with the bacteria and their toxins. For example, the 512 μgml^-^^1^ BF-AuNPs inhibited the *S. aureus, P. aeruginosa*, and *K. pneumoniae*. The same concentration each of BL, NF, ML and OP-C-AuNPs inhibited the growth of *P. aeruginosa, K. pneumoniae* and *S. aureus*, respectively (Figure 9). But with *S. aureus, K. pneumoniae* and others the 600 μgml^-1^ BL-C-AuNPs inhibited the growth. The growth of *S. aureus* and *K. pneumoniae* pathogenic bacteria was inhibited at 512 μgml^-1^ ML-C-AuNPs, the 512 μgml^-1^ OP-C-AuNPs inhibited the growth of *S. aureus*. The ≥512 μgml^-1^ C-AuNPs inhibited the growth of Gram*-*positive and Gram-negative bacteria. In order to determine the MIC of C-AuNPs for antibacterial activities, the C-AuNPs with definite interacting activities attain a bacterial killing maximum ability with its definite population, in other words, from 512 to 600 μgml^-1^. These are expressed as per available chemical activities and nature of bacterial materials, which are intrinsic values and not the normal values obtained out of a wider range from 64 to 1024 μgml^-1^.

A maximum growth inhibition was recorded at 512 μgml^-1^ but some of them were at 600 μgml^-1^. The 64–1024 μgml^-1^ were standardized for growth inhibition of pathogenic bacteria by determining MIC. Although these MIC values are higher, at the same time these values have been directive indicators for successfully materializing our hypothesis. These success indicators establish a research methodology for reducing and simultaneous capping of AuNPs out of HAuCl_4_ precursor contrary to chemical routes. So, our experiments with natural resources for estimation of antibacterial activities without further capping with more functional molecules have been successful for opening a new window in nanoscience and nanotechnology by making a functional use of natural resources in collaboration with surfaces of metallic NPs. Apart from a bare capping with chosen PEs, we have designed studies to further move to the second stage  either to separate out the chemical agents, to allow them to cap the AuNPs or to sort them out for further lowering the MIC as per SAR and friccohesity approaches. These approaches with stronger binding for cohesive forces do not allow the C-AuNPs to strengthen the interaction with the cell wall or other cell organelles and hence needed larger C-AuNPs amounts with higher MIC. Thus, the higher concentrations have opened a direction for further improvement, lowering these concentrations to inhibit the growth of pathogenic bacteria.

## Conclusion

Chemical agents present in chosen plant parts reduced HAuCl_4_ to AuNPs and successfully capped the AuNPs as new C-AuNPs nanomaterials. The C-AuNPs expressed 512 and 600 μgml^-1^ MICs for visible antibacterial activity against *S. aureus, P. aeruginosa* and *K. pneumoniae*. The >512 μgml^-1^ inhibited pathogenic Gram-positive and Gram-negative bacteria up to 99%. The analyses inferred that the C-AuNPs had interacted with the bacterial cell wall due to phytochemicals present in PEs. The C-AuNPs ruptured the cell wall, disturbing the metabolism of bacteria by inducing chemical activities. C-AuNPs could enter inside pathogenic bacteria to destroy the outer cell wall for their interactions with mitochondria and other organelles of bacteria. The chosen plant parts have functional chemical agents to reduce Au^+^ and simultaneously cap for better stability of C-AuNPs.

## Future perspective

Our newly developed green route for reduction and capping of AuNPs could further be applied for lanthanides, TiO_2_, ZnO, MgO and other NPs with varieties of plants and their parts. Determination of their antimicrobial activities for several pathogenic bacteria could establish a relationship between the plant sources *vis-à-vis* metallic NPs for evolving the functional interface. The selective or a preferential tendency of capped NPs with specific cell constituents could be evolved, perhaps for a direct application of such plant parts in remote areas of the human habitats. Apart from pathogenic killing, our green route could also be of great importance to separate out the valuable metallic NPs from mixtures of their metallic NPs because the specific chemical agents of plant parts could approach specific surface areas of the metallic NPs. MICs from ≥512 to 600 μgml^-1^, which inhibited the growth of pathogenic bacteria, could further be reduced using several PEs for capping AuNPs of approximately 20–30 nm size. Such studies under the framework of long-term therapeutic effects could cure chronic diseases. C-AuNPs have suitable activities for controlled drug delivery, cancer treatment, biomedical imaging and others Table 2.

**Table 2.** **Gram-positive and Gram-negative pathogenic bacteria used for antibacterial study.**

**Sr. No.**	**Pathogenic bacteria**	**Nature of bacteria**
1.	*Staphylococcus aureus*	Gram-positive

2.	*Pseudomonas aeruginosa*	Gram-negative

3.	*Klebsiella pneumoniae*	Gram-negative

**Summary points**

- Flower, leaf and peel of plant extracts behaved as reducing agents for gold nanoparticles (AuNPs).
- *In situ* capping was synergized with green AuNP synthesis preventing chemical reducing agents.
- Our methodology was adopted at normal temperature and pressure and gave 96% yields in reducing and capping AuNPs simultaneously.
- A single step synthesis has produced AuNPs of ≈20–30 nm size estimated with transmission electron microscopy, atomic force microscopy and dynamic light scattering.
- Unlike chemical methods, our green methodology produced thermodynamically stable AuNPs with sufficient surface area.
- C-AuNPs have expressed antibacterial activity from 500 to 600 μgml^-1^ MIC, inhibiting pathogenic bacterial growth.
- MIC from 512 to 600 μgml^-1^ inhibited growth of pathogenic bacteria and could further be reduced through research using several plants.

## References

[1] Hobman JL, Wilson JR, Brown NL, Lovely DR (2000). Microbial mercury reduction. *Environmental Metal-Microbe Interactions*.

[2] Inwati GK, Rao Y, Singh M (2016). *In situ* free radical growth mechanism of platinum nanoparticles by microwave irradiation and electrocatalytic properties. *Nanoscale Res. Lett.*.

[3] Lewis K, Klibanov AM (2005). Surpassing nature: rational design of sterile-surface materials. *Trends Biotechnol.*.

[4] Jain S, Hirst DG, O'Sullivan JM (2012). Gold nanoparticles as novel agents for cancer therapy. *Br. J. Radiol.*.

[5] Misbahi A (2010). A review on gold nanoparticles radio sensitization effect in radiation therapy of cancer. *Rep. Pract. Oncol. Radiother.*.

[6] Yugang SUN, Changhua AN (2011). Shaped gold and silver nanoparticles. *Front. Mater. Sci.*.

[7] Zhang Z, Ross RD, Roeder RK (2010). Preparation of functionalized gold nanoparticles as a targeted x-ray contrast agent for damaged bone tissue. *Nanoscale*.

[8] Capek I (2013). Preparation and functionalization of gold nanoparticles. *J. Surf. Sci. Technol.*.

[9] Li J, Li Q, Ma X (2016). Biosynthesis of gold nanoparticles by the extreme bacterium *Deinococcus radiodurans* and an evaluation of their antibacterial properties. *Int. J. Nanomed.*.

[10] Das A, Chadha R, Maiti N, Kapoor S (2014). Role of surfactant in the formation of gold nanoparticles in aqueous medium. *J. Nanopart.*.

[11] Johan MR, Chong LC, Hamizi NA (2012). Preparation and stabilization of monodisperse colloidal gold by reduction with monosodium glutamate and poly (methyl methacrylate). *Int. J. Electrochem. Sci.*.

[12] Abdelhalim MAK, Mady MM, Ghannam MM (2012). Physical properties of different gold nanoparticles: ultraviolet-visible and fluorescence measurements. *J. Nanomed. Nanotechol.*.

[13] Kumar RV, Babu GS, Chauhan S, Srivastava A, Rao Y, Kumar D (2012). Total phenolics and flavonoids content in ripened and unripened fruits of different mulberry (*Morus alba*) varieties. *Indian J. Agric. Sci.*.

[14] Amendola V, Meneghetti M (2009). Size evaluation of gold nanoparticles by UV-Vis spectroscopy. *J. Phys. Chem. C.*.

[15] Prema P, Thangapandiyan S (2013). *In-vitro* antibacterial activity of gold nanoparticles capped with polysaccharide stabilizing agents. *Int. J. Pharm. Pharm. Sci.*.

[16] Klaus T, Joerger R, Olsson E, Granqvist CG (1999). Silver based crystalline nanoparticles, microbially fabricated. *Proc. Natl Acad. Sci. USA*.

[17] Youssef AM, EL-Sayed SM, Salama HH, EL-Sayed HS, Dufresne A (2015). Evaluation of bionanocomposites as packaging material on properties of soft white cheese during storage period. *Carbohydr. Polym.*.

[18] Youssef AM, Abdel-Aziz MS, El-Sayed SM (2014). Chitosan nanocomposite films based on AgNP and AuNP biosynthesis by *Bacillus subtilis* as packaging material. *Int. J. Biol. Macromol.*.

[19] Lansdown ABG, Sampson B, Laupattarakasem P, Vuttivirojana A (1997). Silver aids healing in the sterile skin wound: experimental studies in the laboratory rat. *Br. J. Dermatol.*.

[20] Guerra R, Lima E, Viniegra M, Guzmán G, Lara V (2012). Growth of *Escherichia coli* and *Salmonella typhi* inhibited by fractal silver nanoparticles supported on zeolitas. *Micropor. Mesopor. Mater.*.

[21] Sabbani S, Gallego-Perez D, Nagy A, Waldman J, Hansford D, Duttamcfe PK (2010). Synthesis of silver-zeolite films on micropatterned porous alumina and its application as an antimicrobial substrate. *Micropor. Mesopor. Mater.*.

[22] Haruta M, Tsubota S, Kobayashi T, Kageyama H, Genet M, Delmon B (1993). Low temperature oxidation of CO over gold supported on TiO2, α-Fe2O3, and Co3O4. *J. Catal.*.

[23] Huang J, Lima E, Akita T (2011). Propene epoxidation with O2 and H2: identification of the most active gold clusters. *J. Catal.*.

[24] Day RO, Furst DE, Van Riel PL, Bresnihan B, Michael J, Parnham S (2005). Progress in inflammation research, antirheumatic therapy. *Actions and Outcomes*.

[25] Weidauer E, Yasuda Y, Biswal BK, Cherny M, James MNG, Brömme D (2007). Effects of disease-modifying anti-rheumatic drugs (DMARDs) on the activities of rheumatoid arthritis-associated cathepsins K and S. *Biol. Chem.*.

[26] Arceci RJ (2008). When T cells and macrophages do not talk: the hemophagocytic syndromes. *Curr. Opin. Hematol.*.

[27] Lima E, Guerra R, Lara V, Guzmán A (2013). Gold nanoparticles as efficient antimicrobial agents for *Escherichia coli* and *Salmonella typhi*. *Chem. Cent. J.*.

[28] Zhou Y, Kong Y, Kundu S, Cirillo JD, Liang H (2012). Antibacterial activities of gold and silver nanoparticles against *Escherichia coli* and bacillus Calmette–Guérin. *J. Nanobiotechnol.*.

[29] Youssef AM, Abdel-Aziz MS (2013). Preparation of polystyrene nanocomposites based on silver nanoparticles using marine bacterium for packaging. *Polym. Plast. Technol. Eng.*.

[30] Jorgensen JH, Turnidge JD, Murray PR, Baron EJ, Jorgensen JH, Landry MLP, Faller MA (2007). Susceptibility test methods: dilution and disk diffusion methods. *Manual of Clinical Microbiology (Volume II)*.

[31] Liu FK, Huang PW, Chu TC, Ko FH (2005). Gold seed-assisted synthesis of silver nanomaterials under microwave heating. *Mater. Lett.*.

[32] Ahmada T, Wania IA, Manzoorb N, Ahmed J, Asirid AM (2013). Biosynthesis, structural characterization and antimicrobial activity of gold and silver nanoparticles. *Colloids Surf. B Biointerfaces*.

[33] Ahmad T, Wani IA, Lone IH (2013). Antifungal activity of gold nanoparticles prepared by solvothermal method. *Mater. Res. Bull.*.

[34] Das RK, Borthakur BB, Bora U (2010). Green synthesis of gold nanoparticles using ethanolic leaf extract of *Centella asiatica*. *Mater. Lett.*.

[35] Hernández-Sierra JF, Ruiz F, Pena DC (2008). The antimicrobial sensitivity of *Streptococcus mutans* to nanoparticles of silver, zinc oxide, and gold. *Nanomedicine*.

[36] Zhou Y, Kong Y, Kundu S, Cirillo JD, Liang H (2012). Antibacterial activities of gold and silver nanoparticles against *Escherichia coli* and bacillus Calmette–Guérin. *J. Nanobiotechnol.*.

[37] Tarnawski R, Ulbricht M (2011). Amphiphilic gold nanoparticles: synthesis, characterization and adsorption to PEGylated polymer surfaces. *Colloids Surf. A Physicochem. Eng. Aspects*.

[38] Lazos DS, Ulbricht FM (2005). Size-selective protein adsorption to polystyrene surfaces by self-assembled grafted poly(ethylene glycols) with varied chain lengths. *Langmuir*.

[39] Shamaila S, Zafar N, Riaz S, Sharif R, Nazir J, Naseem S (2016). Gold nanoparticles: an efficient antimicrobial agent against enteric bacterial human pathogen. *Nanomaterials*.

[40] Oyewole OA, Abalaka ME (2012). Antimicrobial activities of *Telfairia occidentalis* (fluted pumpkins) leaf extract against selected intestinal pathogens. *J. Health Sci.*.

[41] Chandran K (2014). Effect of size and shape controlled biogenic synthesis of gold nanoparticles and their mode of interactions against food borne bacterial pathogens. *Arab. J. Chem.*.

