# Supplementary material for: Green synthesis of capped gold nanoparticles and their effect on Gram-positive and Gram-negative bacteria
Source: Future Sci OA. 2017 Sep 5;3(4):FSO239. doi: 10.4155/fsoa-2017-0062 (PMC5674245; doi:10.4155/fsoa-2017-0062)
Supplement: Supplementary file 1 [file fsoa-03-239-s1.docx]

**Supplementary Information (SI)**

**Green Synthesis of capped Au NPs and their effect on Gram–Positive and Gram–negative Bacteria**

Yashvant Rao^1^, Gajendra K. Inwati^1^, Man Singh^2^

1, Centre for Nanosciences,

2, School of Chemical Sciences

Central University of Gujarat, Gandhinagar, Gujarat, India

**The chemical composition of plant extracts:**

These are the basic and very essential chemical components present in the PE

***BF and BLE, O**leanolic acid, Ursolic acid, Rosmarinic acid, ^1^ Eugenol, Carvacrol, Linalool, β–caryophyllene (about 8%), ^2^ β–element (c.11.0%), and Gacreneerm D (about 2%)^3^.

****NFE** Behenic (0.7%), Arachidic (0.7%), Stearic (8.2%), Palmitic (13.6%), Oleic (6.5%) and Linoleic (8.0%) ^4^

**#MLE** menthol (7–48%), menthone (20–46%), menthyl acetate (3–10%), menthofuran (1–17%) and 1,8-cineol (3–6%). Peppermint oil also contains small amounts of many additional compounds including limonene, pulegone, caryophyllene and pinene.^5^.

**##OPE** Limonene, α-Pinene, Sabinene, Myrcene, Octanal, Linalool, δ-3-Carene and Decanal ^6^.

**NOTE:** (* Basil Flower and Basil Leaves Extracts, **Neem Flower Extracts, # Mentha Leaves Extracts, and ## Orange Peel Extracts)

**Chemical composition of Basil**

**SI Figure 1 (a)**

**Chemical composition of NEEM flower extract**

**SI Figure 1 (b)**

**Chemical composition of MENTHA leaves extract**

**SI Figure 1 (c)**

**Chemical composition of ORANGE PEEL extract**

**SI Figure 1 (d)**

**(SI Table 1)**

**DLS study of the Green synthesized Au NPs like SD, PDI, ZP, PO, CON, VIS etc.**

|  | **BFGP** | | **BLGP** | **NFGP** | **MLGP** | **OPGP** |
| --- | --- | --- | --- | --- | --- | --- |
| **SD** | 6.97 | 21.07 | | 288.8 | 42.3 | 8.87 |
| **PDI** | 3.77 | | 0.0582 | 3.77 | 0.2846 | 2.502 |
| **ZP (mV)** | -3.86 | | -0.59 | 3.86 | -0.58 | -0.54 |
| **PO** | Negative | | Negative | Positive | Negative | Negative |
| **CON (uS/cm)** | 143 | | 915 | 143 | 720 | 813 |
| **VIS (cp)** | 0.97 | | 0.839 | 0.97 | 0.836 | 0.776 |

**AFM Study of Green synthesized Au NPs** essential information of size, height, roughness and 3D topographical image with size due to their natural properties of PE extracts respectably. The SI Table 2 represents this information of a graph having different size and morphology with a histogram of Au NPs.

**(SI Table 2)**

|  | Min (nm) | Max  (nm) | Mid (nm) | Mean (nm) | Rpv (nm) | Rq (nm) | Ra (nm) | Rz (nm) | Rsk | Rku |
| --- | --- | --- | --- | --- | --- | --- | --- | --- | --- | --- |
| MLGP | -0.602 | 1.126 | 0.262 | 0.063 | 1.728 | 0.416 | 0.309 | N/A | -0.77 | 3.697 |
|  | -0.47 | 0.686 | 0.108 | 0.022 | 1.156 | 0.31 | 0.245 | 0.712 | -0.357 | 2.573 |
| NFGP | -0.46 | 1.37 | 0.455 | 0.158 | 1.83 | 0.506 | 0.386 | N/A | -1.35 | 3.634 |
|  | -0.254 | 1.14 | 0.443 | 0.157 | 1.393 | 0.441 | 0.371 | N/A | 1.133 | 2.787 |
| BFGP | -0.648 | 0.518 | -0.065 | -0.025 | 1.166 | 0.257 | 0.191 | N/A | -0.149 | 3.465 |
|  | 0.613 | 0.484 | -0.065 | -0.014 | 1.097 | 0.256 | 0.195 | N/A | 0.287 | 3.123 |
| BLGP | 0.195 | 1.493 | 0.844 | 0.719 | 1.298 | 0.288 | 0.205 | 0.507 | -0.869 | 3.93 |
|  | 0.466 | 1.56 | 1.013 | 0.893 | 1.094 | 0.294 | 0.238 | 0.309 | -0.795 | 2.893 |
| OPGP | -0.493 | 0.631 | 0.069 | 0.084 | 1.124 | 0.356 | 0.283 | N/A | 0.454 | 2.223 |
|  | -0.552 | 0.669 | 0.059 | 0.035 | 1.222 | 0.384 | 0.322 | N/A | 0.083 | 1.947 |

SI table 3 Antibacterial study of *K. pneumoniae* with Au NPs at different concentration

| ***K. pneumoniae*** | | | | | |
| --- | --- | --- | --- | --- | --- |
| µgmL^-1^ | BF Au NPs | BL Au NPs | NF Au NPs | ML Au NPs | OP Au NPs |
| 1024 | - | - | - | - | - |
| 900 | - | - | - | - | - |
| 800 | - | - | - | - | - |
| 700 | - | - | - | - | - |
| 600 | - | - | - | - | - |
| 512 | - | + | - | - | + |
| 256 | + | + | + | + | + |
| 128 | + | + | + | + | + |
| 64 | + | + | + | + | + |
| MIC | >512 | >512 | >512 | >512 | >512 |
| MIC Value | 512 | 600 | 512 | 512 | 600 |
| **+ means growth of microbial strain; ‐ means no growth of microbial strains** | | | | | |

SI table 4 Antibacterial study of *P. aeruginosa* with Au NPs at different concentration

| ***P. aeruginosa*** | | | | | |
| --- | --- | --- | --- | --- | --- |
| µgmL-1 | BF Au NPs | BL Au NPs | NF Au NPs | ML Au NPs | OP Au NPs |
| 1024 | - | - | - | - | - |
| 900 | - | - | - | - | - |
| 800 | - | - | - | - | - |
| 700 | - | - | - | - | - |
| 600 | - | - | - | - | - |
| 512 | - | - | + | + | + |
| 256 | + | + | + | + | + |
| 128 | + | + | + | + | + |
| 64 | + | + | + | + | + |
| MIC | >512 | >512 | >512 | >512 | >512 |
| MIC Value | 512 | 512 | 600 | 600 | 600 |
| **+ means growth of microbial strain; ‐ means no growth of microbial strains** | | | | | |

SI table 4 Antibacterial study of *S. aureus* with Au NPs at different concentration

| ***S. aureus*** | | | | | |
| --- | --- | --- | --- | --- | --- |
| µgmL-1 | BF Au NPs | BL Au NPs | NF Au NPs | ML Au NPs | OP Au NPs |
| 1024 | - | - | - | - | - |
| 900 | - | - | - | - | - |
| 800 | - | - | - | - | - |
| 700 | - | - | - | - | - |
| 600 | - | - | - | - | - |
| 512 | - | + | + | - | - |
| 256 | + | + | + | + | + |
| 128 | + | + | + | + | + |
| 64 | + | + | + | + | + |
| MIC | >512 | >512 | >512 | >512 | >512 |
| MIC Value | 512 | 600 | 600 | 512 | 512 |
| **+ means growth of microbial strain; ‐ means no growth of microbial strains** | | | | | |

**References of the chemical composition of plant extracts:**

1. R. Shanmuga Sundaram, M. Ramanathan, R. Rajesh, B. Satheesh & D. Saravanan LC-MS quantification of Rosmarinic acid and Ursolic acid in the Ocimum Sanctum Linn. Leaf Extract (Holy Basil, Tulsi), *Journal of Liquid Chromatography & Related Technologies*, 2012; 35 (5) 634-650. DOI: 10.1080/10826076.2011.606583
2. Kuhn, Merrily; David Winston *Winston & Kuhn's Herbal Therapy & Supplements: A Scientific and Traditional Approach*. Lippincott Williams & Wilkins. p. 260. 2007; ISBN:978-1-58255-462-4.
3. Padalia, Rajendra C., Verma, Ram S., "Comparative volatile oil composition of four *Ocimum* species from northern India". *Natural Product Research*. 2011; **25** (6) 569–575. DOI:10.1080/14786419.2010.482936. PMID 21409717
4. http://www.neemfoundation.org/about-neem/chemistry-of-neem/
5. Leung, A. Y. (1980). Encyclopedia of Common Natural Ingredients used in food, drugs, and cosmetics. *New York: John Wiley & Sons. p*. 231
6. Steinke, K., Jose, E., Sicker, D., Siehl, H.-U., Zeller, K. P. Berger, S., Sinensetin. Chemie in unserer Zeit. 2013; 47: 158–163. DOI:10.1002/ciuz.201300627
